# Exploring the relationships between resilience, burnout, work engagement, and intention to leave among nurses in the context of the COVID-19 pandemic: a cross-sectional study

**DOI:** 10.1186/s12912-024-01958-1

**Published:** 2024-04-29

**Authors:** Apiradee Nantsupawat, Ann Kutney-Lee, Kulwadee Abhicharttibutra, Orn-Anong Wichaikhum, Lusine Poghosyan

**Affiliations:** 1 Faculty of Nursing, Chiang Mai University, Chiang Mai, Thailand; 2 Center for Health Outcomes and Policy Research, University of Pennsylvania School of Nursing, Philadelphia, USA; 3 Columbia University School of Nursing, New York, USA

**Keywords:** Resilience, Burnout, Intention to leave, Work engagement, Thailand, COVID-19 pandemic

## Abstract

**Background:**

Nurses have faced significant personal and professional stressors during the COVID-19 pandemic that have contributed to increased rates of burnout, intention to leave, and poorer work engagement. Resilience has been identified as a critical factor influencing job outcomes; however, the dynamics of this association have not yet been investigated within the context of the Thai workforce. The study objective was to determine the associations between resilience and job outcomes, including burnout, intention to leave, and work engagement among nurses working in Thailand during the COVID-19 pandemic.

**Methods:**

This cross-sectional study gathered data from a sample of 394 registered nurses employed across 12 hospitals. The research instruments comprised the Connor–Davidson Resilience Scale (CD-RISC), the Maslach Burnout Inventory-Health Services Survey (MBI-HSS), a questionnaire assessing the intention to leave the job, and the Utrecht Work Engagement Scale (UWES). To determine the associations among the measured variables, multivariate logistic regression analyses were conducted.

**Results:**

One-third of nurses experienced emotional exhaustion and depersonalization, and about half experienced reduced personal accomplishment; one-tenth of nurses intended to leave their job. Nurses who exhibited higher levels of resilience were found to have a significantly reduced likelihood of experiencing high emotional exhaustion, depersonalization, and a diminished sense of personal accomplishment. Conversely, these nurses were more likely to report higher levels of work engagement than their less resilience.

**Conclusion:**

The COVID-19 pandemic offers important lessons learned about promoting the well-being of the nursing workforce and protecting against adverse job outcomes. While we identified resilience as a significant predictor of several nurse outcomes, other work environment factors should be considered. Government and hospital administrations should allocate resources for individual and organizational-level interventions to promote resilience among frontline nurses so that hospitals will be better prepared for the next public health emergency and patient and nurse outcomes can be optimized.

**Supplementary Information:**

The online version contains supplementary material available at 10.1186/s12912-024-01958-1.

## Background

Since the coronavirus disease 2019 (COVID-19) global health emergency was declared in January 2020 [6], nurses working on the frontlines have faced substantial professional and personal stress. Especially during the early phases of the pandemic, nurses working in hospitals experienced staffing shortages and worked long hours with limited medical supplies and resources, all while placing their personal health and well-being at risk [3]. Three years later, the pandemic has eased due to effective vaccines and treatments; however, the pandemic’s impact on the nursing workforce is still being felt. The adverse effects of working during the pandemic on nurses’ physical (e.g., fatigue, sleeplessness) and psychosocial (e.g., post-traumatic stress disorder, depression) well-being have been well-documented [7]. Taken together, these stressors are increasingly acknowledged as significant contributors to increasing rates of burnout and intention to leave and lower levels of work engagement among nurses, all of which may negatively affect patient safety [21].

Numerous international studies, including systematic reviews and meta-analyses, have documented higher rates of burnout [15] and intention to leave [41] among nursing professionals in the period of the COVID-19 pandemic. Burnout is defined as a syndrome emerging from a context of unmanaged chronic workplace stress. Burnout is characterized by three core dimensions: a sustained sense of exhaustion and energy loss, a growing detachment or a cynical attitude toward one’s job, and a perceived decline in professional efficacy and accomplishment [44]. Employing the three distinct subscales of the MBI—a widely recognized tool for assessing burnout—Galanis et al. [15] found that 34.1% exhibited substantial emotional exhaustion in their global analysis of nurses. Additionally, 12.6% demonstrated significant levels of depersonalization, while 15.2% reported a profound lack of personal accomplishment.

Similarly, nurses also had high rates of intention to leave, referred to as one’s plan to leave their current job, and is strongly related to actual turnover [17]. The construct of ‘intention to leave’ is widely utilized as a predictive indicator of the potential attrition within the nursing workforce, which has significant implications for maintaining safe workplace environments and ensuring the quality of patient care [23]. A recent systematic review and meta-analysis to evaluate the prevalence of turnover intentions among nurses throughout the COVID-19 pandemic revealed that approximately 31.7% of nurses were considering leaving their positions during the outbreak [41]. Moreover, turnover intentions were higher than before COVID-19 [33].

Work engagement among nurses has also been impacted by the pandemic [2, 10]. Work engagement is commonly conceptualized as a positive and fulfilling work-related state of mind that is manifested by three key elements: vigor, dedication, and absorption [37]. Vigor is defined as the propensity to expend persistent effort in one’s occupational tasks; dedication encompasses the degree of personal involvement in one’s professional role; and absorption pertains to the depth of concentration and engrossment in work-related activities. Nurses with high levels of engagement can work in extraordinarily stressful conditions and handle undesirable consequences while continuing to contribute to work-related creativity, productivity, and job performance. Past research has demonstrated that a workforce composed of highly engaged nurses is associated with high-quality patient care [34, 39]. Research on work engagement among nurses internationally during the COVID-19 pandemic has yielded inconsistent results. Some studies have identified low to moderate levels of work engagement among frontline nurses during the pandemic [10, 16, 28], whereas other studies report high levels of engagement [2, 26]. Differences in work engagement among nurses have been attributed to differences in resilience, which may be influenced by both personal attributes and the environment in which nurses work [35].

Research is needed to understand factors that reduce burnout and intention to leave and increase work engagement among nurses to promote workforce retention and maintain quality of care throughout the pandemic and beyond. Such evidence is critically important for healthcare administrators and policymakers to invest efforts to support the nursing workforce in critical times. With innovative policies supporting nurses’ well-being, patients may experience better care. During the COVID-19 pandemic, studying nurse resilience has emerged as an increasingly studied characteristic of nurses that may be essential to these outcomes. Resilience is defined as the capacity to recover from difficulties or adapt to challenging and adverse circumstances [13]. An individual demonstrating resilience actively endeavors to progress constructively, drawing upon the insights and knowledge gained from confronting adverse experiences. Indeed, several studies among healthcare workers and nurses during the pandemic have found associations between resilience and lower levels of burnout [4, 20, 25, 42], intention to leave [29, 40] and a higher level of work engagement [9, 26]. However, these studies have been limited to the United States, Australia, China, Brazil, and Spain. Furthermore, many of these studies focused on resilience without adequate emphasis on significant contextual factors necessary for safe nursing care.

Thailand was recognized as the first country to confirm cases of COVID-19 beyond the borders of the People’s Republic of China. The preliminary measures enacted by the Thai Ministry of Public Health to mitigate the spread of the pandemic were centered on enhancing personal and communal hygiene practices [45]. Following the proclamation of an emergency decree, the Thai government implemented stringent control measures to curtail the spread of COVID-19. These measures included the imposition of travel restrictions, the prohibition of public gatherings, the enforcement of physical distancing, the strategic lockdown of areas deemed high-risk for transmission, the suspension of all international flights into Thailand, and mandatory government-managed quarantine for individuals returning from abroad for 14 days (Department of Disease Control, 2020). When this study was conducted in 2022, a cumulative total of 4.7 million COVID-19 cases had been confirmed in Thailand. During the height of the pandemic, however, resources to support nurses and other healthcare professionals in the delivery of patient care were under severe constraints [24]. As such, nurses in Thailand have experienced burnout, depression, and post-traumatic stress disorder [8] at rates similar to those documented in other countries [15]. However, to our knowledge, studies have yet to be conducted on nurse resilience and its relationships with nurse job outcomes in Thailand. This study examined the associations between resilience and job outcomes, including burnout, intention to leave, and work engagement among nurses working in Thailand during the COVID-19 pandemic.

## Methods

### Participants and procedures

The sample size was determined using the Yamane formula (1973), with a 95% confidence level and *p* = 0.05. Including an allowance of approximately 20% for potential loss, 477 questionnaires were distributed to nurses who were required to possess a minimum of one year of experience at general hospitals, providing direct nursing care to patients during the COVID-19 pandemic. A multistage sampling method was conducted to enroll participants from 12 general hospitals with 200–500 beds nationwide located in six of the country’s twelve health regions. This investigation constitutes a segment of a study examining nurses’ perceptions of job-related outcomes amidst the COVID-19 pandemic in Thailand’s general hospitals. For further details regarding the sample selection, please look at a prior study [32].

Before starting data collection, ethical approval was obtained from the Research Ethics Review Committee of the Faculty of Nursing under the reference number EXP:055-2022 and by the hospital administrators of the participating hospitals. Then the researcher convened with coordinators who were hospital staff designated by the hospital administrators to collect the data and to explain data collection procedures. Invitations and questionnaires were sent to nurses. Nurses who indicated a willingness to participate were issued invitations detailing the study’s objectives, the methodologies employed in data collection, the estimated time commitment required for questionnaire completion, the potential risks and benefits associated with the study, and guarantees of participant confidentiality. Participants were instructed to return the completed questionnaires and signed informed consent documents in separate, sealed envelopes to a designated collection point. Hospital coordinators were responsible for forwarding all collected materials to the principal investigator via postal service. Of the 477 questionnaires distributed to nurses who met the inclusion criteria, 394 were completed and returned, yielding a response rate of 82.6%.

### Instruments

The questionnaire included demographic items and measures of resilience, burnout, intent to leave, and work engagement. Demographic items included age, gender, education, marital status, years working on their current unit, years in professional nursing, the number of children, and family members.

#### Resilience

We used the CD-RISC-10, a 10-item instrument, to measure resilience. The items were scored on a 5-point Likert scale ranging from 0 (never) to 4 (almost always), with higher scores reflecting greater resilience [11]. The Cronbach’s alpha coefficient for the instrument was calculated to be 0.92 in the current study.

*Burnout*: We used the Maslach Burnout Inventory-Health Services Survey (MBI-HSS) [27] to measure burnout. The three subscales of the MBI-HSS include Emotional Exhaustion (EE), Depersonalization (DPER), and Personal Accomplishments (PAC) and consist of nine items, five items, and eight items, respectively. Responses are evaluated using a 7-point Likert scale ranging from 0, indicating ‘never’, to 6, signifying ‘every day’. The following criteria can characterize burnout: achieving a score of 27 or higher on the Emotional Exhaustion (EE) subscale, attaining a score of 10 or higher on the Depersonalization (DPER) subscale, and scoring below 33 on the Personal Accomplishment (PAC) subscale. We have obtained the permission to use this scale. The MBI-HSS has been translated into Thai and was utilized in a prior study conducted in Thailand. The Cronbach’s alpha for the three MBI-HSS subscales were 0.89, 0.79, and 0.87, respectively [31].

#### Intention to leave

On the questionnaire, respondents were asked if they intended to leave their current position in the next year. Responses included leaving the job within 6 or 12 months and without intention to leave. The intention of nurses to leave has been demonstrated to serve as a predictor for the subsequent decision to leave the position, and it can also serve as an early indicator of potential turnover [14].

#### Work engagement

We used the UWES-9, a 9-item instrument with three subscales, vigor, dedication, and absorption, to measure work engagement. The instrument is scored on a 7-point Likert-type scale ranging from never (1) to always (7) [37]. A higher score indicates better work engagement. Following the scoring guidance provided by Schaufeli and Bakker [37], nurses with a UWES-9 score equal to or above the 75th percentile were defined as having “high work engagement.” In the current study, the internal consistency of the UWES-9 was assessed using Cronbach’s alpha, yielding a coefficient of 0.92, which indicates excellent reliability within our sample.

### Data analysis

Descriptive statistics were calculated to examine the characteristics of nurses and key variables of interest, including resilience, burnout, intention to leave, and work engagement. Logistic regressions were applied to explore the association of resilience with work engagement, burnout, and intention to leave. These associations were estimated unadjusted and then adjusted for nurse characteristics including age, gender, education, marital status, years worked as a RN, and the number of family members. We used the Huber-White sandwich estimator to adjust for the clustering of nurses within hospitals. All tests were performed using 0.05 levels of significance. Data analyses were performed using STATA software, version 14 (StataCorp, 2015).

## Results

Table 1 presents the characteristics of nurses in the sample. Nurses were 37 years of age on average, while most were female (95.18%) and held a bachelor’s degree (90.61%). Over half of the sample (52.28%) were married, and over 76.84% had at least one child. Over half (51.52%) of the participants lived with more than three family members. On average, nurses had worked for nearly 14 years as registered nurses and nine years in their current unit. The mean resilience score among nurses in the sample was 28.07 (S.D.= 7.20). The assessment of burnout using the three MBI-HSS subscales yielded the following results: 35.79% of nurses reported high levels of emotional exhaustion, 29.95% reported high levels of depersonalization, and 48.22% reported diminished personal accomplishment. Nearly one in ten nurses (9.14%) indicated an intention to leave their current job within 6 or 12 months, and over one-quarter (27.41%) reported a high level of work engagement.

**Table 1:** Characteristics of nurses

Variables	Mean	SD	Frequency	%
Age	37.45	10.04		
Gender				
Male			19	4.82
Female			375	95.18
Education				
Bachelor’s degree			357	90.61
Higher than a bachelor’s degree			37	9.39
Marital status				
Single			188	47.71
Married			206	52.28
Number of Children				
0			44	23.16
>1			146	76.84
Number of family members				
0			77	19.54
1–2			114	28.94
>3			203	51.52
Years worked as RN	13.95	9.55		
Years working in the current unit	9.49	7.79		
Connor–Davidson Resilience Scale-10	28.07	7.20		
MBI-HSS: Emotional Exhaustion subscale	23.28	9.82		
High emotional exhaustion (≥ 27)			141	35.79
MBI-HSS: Depersonalization subscale	6.72	6.10		
High depersonalization (≥ 10)			118	29.95
MBI-HSS: Personal Accomplishment subscale	32.46	10.44		
Reduced personal accomplishment (≤ 33)			190	48.22
Intention to leave in current job				
Yes, within 6 or 12 months			36	9.14
Not decided			358	90.86
Utrecht Work Engagement Scale-9	43.84	11.12		
High work engagement (≥ 75 percentile)			108	27.41

Notes: MBI-HSS = Maslach Burnout Inventory-Health Services Survey; RN = registered nurse

Table 2 displays the results of the unadjusted and adjusted logistic regression models indicating the effect of a one-unit increase in the resilience score on the odds of burnout (as indicated by high emotional exhaustion, high depersonalization, and reduced personal accomplishment), intention to leave, and work engagement. In the unadjusted models, higher levels of resilience demonstrated statistically significant associations with all five outcomes. In the adjusted models that accounted for individual differences in age, gender, highest education, years worked as RNs, marital status, and the number of family numbers, a one-unit increase in the resilience score was associated with lower odds of high emotional exhaustion (odds ratio [OR] 0.98, 95% confidence interval [CI]: 0.97–0.99), high depersonalization (OR 0.97, 95% CI: 0.96–0.98), reduced personal accomplishment (OR 0.98, 95% CI: 0.98–0.99). A one-unit increase in the resilience score was associated with a 4% increase in the odds of reporting high work engagement (OR 1.04, 95% CI: 1.03–1.05). The association between higher resilience and intention to leave became statistically insignificant in the adjusted models (*p* = 0.081).

**Table 2:** Odds Ratios Indicating the Effect of Resilence on MBI-HSS Subscales, Intention to Leave, and Work Engagement

Outcomes	Unadjusted			Adjusted		
	OR	95% CI	*p*-value	OR	95% CI	*p*-value
High Emotional Exhaustion	0.98*	(0.97–0.99)	0.014	0.98*	(0.97–0.99)	0.012
High Depersonalization	0.97*	(0.96–0.98)	< 0.001	0.97*	(0.96–0.98)	< 0.001
Reduced Personal Accomplishment	0.98*	(0.98–0.99)	< 0.001	0.98*	(0.98–0.99)	< 0.001
Intention to leave	0.98*	(0.97–0.99)	0.044	0.98	(0.97-1.00)	0.081
High Work Engagement	1.04*	(1.02–1.05)	< 0.001	1.04*	(1.03–1.05)	< 0.001

Notes: MBI-HSS = Maslach Burnout Inventory-Health Services Survey; OR = odds ratio; CI = confidence interval. The MBI-HSS subscales include emotional exhaustion, depersonalization and personal accomplishment. Adjusted model accounted for age, gender, highest education, years worked as registered nurse (RN), marital status, and number of family numbers. *p ≤ 0.05

## Discussion

In this study of Thai nurses conducted during the COVID-19 pandemic, we observed a significant association between resilience and nurse job outcomes related to retention and patient safety. These relationships were observed even after accounting for several individual characteristics, such as age and experience, that could be associated with job outcomes. While the observed level of emotional exhaustion (35.8%) among Thai nurses was similar to the estimated 34.1% reported by Galanis and colleagues [15] in a recent meta-analysis of international studies, levels of high depersonalization and reduced personal accomplishment among nurses in Thailand were markedly higher than other countries (29.6% vs. 12.6% for high depersonalization; 48.2% vs. 15.2% for reduced personal accomplishment). Compared to other countries, the study sample had a higher resilience score than nurses in the Regional Health Service in Spain [36], nurses at the Japanese Red Cross Medical Center in Japan [5], and frontline nurses in Wuhan, China [19]. Higher resilience among Thai nurses may be attributable to growing up in close-knit families and having strong community ties that provided additional emotional and social support through this time of added stress and adversity. In our study, one-tenth of Thai nurses intended to leave their job which was lower compared to other countries where researchers have found that about one-third of nurses intended to leave their job during the COVID-19 pandemic [41]. Although previous studies were undertaken at varying stages of the pandemic, the present research augments the global body of knowledge by providing preliminary comparative data on the resilience and occupational outcomes among nurses in Thailand throughout the COVID-19 crisis.

The results of the current study indicated that higher levels of resilience were associated with lower levels of emotional exhaustion, depersonalization, and diminished personal accomplishment among nurses working in Thailand during the pandemic period. The findings largely confirm previous studies where higher resilience has been linked to lower emotional exhaustion, depersonalization, and reduced personal accomplishment among nurses working in public hospitals in Madrid, Spain [20], nurse managers in tertiary public hospitals in China [25], nurses and nursing students from a college and a state nursing association in Pennsylvania, USA [18], nurses in Brazil [42], and hospital staff in Australia [4].

Our adjusted models did not observe a statistically significant association between resilience and intention to leave. The current findings diverge from those of earlier studies, which demonstrated that an elevated level of resilience among nurses correlated with a reduced propensity to leave their positions. This was particularly evident in studies of nurses employed within a multi-hospital system in the southeastern United States during the COVID-19 pandemic [22] and amongst nurse leaders based in Birmingham, Alabama, USA [29].

A systematic review examining the determinants of turnover intentions among nursing personnel during the COVID-19 pandemic found that resilience could mitigate negative associations with turnover intention [40]. Since our survey was conducted two years into the pandemic, it is possible that nurses who completed the survey were those with higher levels of resilience who persevered through the surges of the pandemic and remained committed to their job.

Nurses with higher resilience may have a well-developed adaptive process to deal with trauma, adversity, tragedy, and stressors [39]. As such, they can embrace problem-solving, cope with emotional avoidance, and pursue social support to persevere through crises. These positive personal characteristics may contribute to nurses being able to work effectively in a traumatic crisis such as COVID-19 [12]. While these nurses may have been exhausted and fatigued during the height of the pandemic, their higher levels of resilience allowed them to cope effectively and, therefore, have lower feelings of burnout.

Finally, our study found that higher levels of resilience were associated with reporting high work engagement. Our findings are similar to previous international studies, which demonstrated that nurses who worked during the pandemic with higher resilience had higher work engagement, including emergency department nurses in Midwestern and Southwestern US states [9] and frontline nurses in 6 provinces of China [26]. Our findings suggest that nurses with resilient personalities working during the pandemic were better positioned to recover from adverse work-related situations. Through an adaptive process, building resilience may have allowed nurses to overcome the crisis due to personal strength, positive psychological adjustment, and the ability to maintain function [39]. Therefore, even though working in stressful events, nurses with higher levels of resilience can continue to be involved at work accompanied by feelings of energy, enthusiasm, and significance.

Although personal characteristics partially determine the development of resilience, the characteristics of the work environment are also recognized as a significant contributing factor [35]. Indeed, nurses across the globe have been practicing in challenging work environments during the COVID-19 pandemic. A preceding study recently established that nurses employed in hospitals characterized by better work environments before the pandemic have reported markedly improved patient care quality and enhanced well-being among clinicians throughout the pandemic [1]. Further, a robust body of evidence from Thailand shows that hospital nurses working in poor work environments are likelier to leave their jobs [30].

## Implications

Even though our study was conducted in one country, our findings have implications for health policies internationally. The COVID-19 pandemic was a global phenomenon, and all frontline nurses have been experiencing similar challenges. Our findings suggest that interventions to build nurse resilience may improve nurse well-being and work engagement, which have been linked to better patient care. At the individual level, the allocation of time for emotional recharge, the availability of peer support, and the encouragement of open communication can facilitate the development and enhancement of an individual’s inherent adaptive systems, thereby fostering resilience [46]. However, other research suggests that resilience training interventions alone may not significantly impact nurse outcomes if not coupled with organizational policies to improve the nurse work environment [43]. In terms of organizational-level actions, hospital and nurse administrators can foster the development of work environments that provide adequate resources and support, promote nurse involvement in organizational decision-making, and promote collegial relationships between nurses and physicians. Further, hospital-based programs that provide positive reinforcement and recognition, coping strategies [38], and training on resilience [46] may also be considered. Such organizational strategies are essential for everyday nursing care but are even more critical during crises such as the COVID-19 pandemic.

### Limitations

Some limitations of the study should be considered while interpreting the results. This study collected data between August 2022 and October 2022, after the COVID-19 pandemic peaked in Thailand; this may influence nurses’ reported levels of resilience. Furthermore, unmeasured confounding variables, such as the quality of the work environment, could introduce bias into our estimates. Future work on resilience and nurse outcomes should incorporate work environment measures. Finally, the potential for same source bias exists in our study as the same nurses provided data for the independent and dependent variables.

## Conclusion

The COVID-19 pandemic has exerted a sustained and profound effect on healthcare systems worldwide for three years. As frontline healthcare professionals during this time, nurses are at high risk for burnout, intention to leave their job, and poor work engagement, which can negatively impact nurse retention and quality of care. Our study is one of the pioneering investigations to examine nurses working during the pandemic in Thailand. We found that higher levels of resilience were a protective factor against burnout and intention to leave and were positively associated with higher work engagement. Nevertheless, resilience should not be considered the only factor necessary for the well-being of nurses. The COVID-19 pandemic offers important lessons learned about many factors that should be improved, including the work environment, to promote the well-being of the nursing workforce and protect against adverse job outcomes. Government and hospital administrations should allocate financial support and resources to develop individual and organizational-level interventions to promote resilience among frontline nurses so that hospitals will be better prepared for the next public health emergency and patient and nurse outcomes can be optimized.

### Electronic supplementary material

Below is the link to the electronic supplementary material.

Supplementary Material 1

Supplementary Material 2

Supplementary Material 3

## Data Availability

All data supporting the findings of this study are available within the paper and its Supplementary Information including study instruments such as the Connor-Davison Resilience Scale-10 (CD-RISC-10) and the Utrecht Work Engagement Scale-9 (UWES-9).

## Abbreviations

CD-RISC: Connor–Davidson Resilience Scale
MBI-HSS: Maslach Burnout Inventory–Health Services Survey
UWES: Utrecht Work Engagement Scale
COVID-19: Coronavirus Disease 2019
EE: Emotional Exhaustion
DPER: Depersonalization
PAC: Personal Accomplishments
