# Supplementary material for: Exploring the relationships between resilience, burnout, work engagement, and intention to leave among nurses in the context of the COVID-19 pandemic: a cross-sectional study
Source: BMC Nurs. 2024 Apr 29;23:290. doi: 10.1186/s12912-024-01958-1 (PMC11057140; doi:10.1186/s12912-024-01958-1)
Supplement: Supplementary file 1 — Supplementary Material 1 [file 12912_2024_1958_MOESM1_ESM.docx]

The Utrecht Work Engagement Scale-9 (UWES-9)

| Items | Never  0 | 1 | 2 | 3 | 4 | 5 | Always  6 |
| --- | --- | --- | --- | --- | --- | --- | --- |
| At my work, I feel bursting with energy |  |  |  |  |  |  |  |
| At my job, I feel strong and vigorous |  |  |  |  |  |  |  |
| When I get up in the morning, I feel like going to work |  |  |  |  |  |  |  |
| I am enthusiastic about my job |  |  |  |  |  |  |  |
| I am proud on the work that I do |  |  |  |  |  |  |  |
| My job inspires me |  |  |  |  |  |  |  |
| I am immersed in my work |  |  |  |  |  |  |  |
| I get carried away when I’m working |  |  |  |  |  |  |  |
| I feel happy when I am working intensely |  |  |  |  |  |  |  |
