# Supplementary material for: Exploring the relationships between resilience, burnout, work engagement, and intention to leave among nurses in the context of the COVID-19 pandemic: a cross-sectional study
Source: BMC Nurs. 2024 Apr 29;23:290. doi: 10.1186/s12912-024-01958-1 (PMC11057140; doi:10.1186/s12912-024-01958-1)
Supplement: Supplementary file 2 — Supplementary Material 2 [file 12912_2024_1958_MOESM2_ESM.docx]

An intention to leave questionnaire.

1.Do you intended to leave your current position?

Yes, within 6 months.

Yes, within 12 months.

No, without intention to leave.
