# Supplementary material for: Exploring the relationships between resilience, burnout, work engagement, and intention to leave among nurses in the context of the COVID-19 pandemic: a cross-sectional study
Source: BMC Nurs. 2024 Apr 29;23:290. doi: 10.1186/s12912-024-01958-1 (PMC11057140; doi:10.1186/s12912-024-01958-1)
Supplement: Supplementary file 3 — Supplementary Material 3 [file 12912_2024_1958_MOESM3_ESM.docx]

The Connor-Davison Resilience Scale-10 (CD-RISC-10)

| Items | Not true at all | Rarely true | Sometimes true | Often true | True nearly all the time |
| --- | --- | --- | --- | --- | --- |
| I am able to adapt when changes occur |  |  |  |  |  |
| I can deal with whatever comes my way |  |  |  |  |  |
| I try to see the humorous side of things when I am faced with problems |  |  |  |  |  |
| Having to cope with stress can make me stronger |  |  |  |  |  |
| I tend to bounce back after illness, injury or other hardships |  |  |  |  |  |
| I believe I can achieve my goals, even if there are obstacles |  |  |  |  |  |
| Under pressure, I stay focused and think clearly |  |  |  |  |  |
| I am not easily discouraged by failure |  |  |  |  |  |
| I think of myself as a strong person when dealing with life’s challenges and difficulties. |  |  |  |  |  |
| I am able to handle unpleasant or painful feelings like sadness, fear, and anger |  |  |  |  |  |
